# Variations of Lipoprotein(a) Levels in the Metabolic Syndrome: A Report from the Maracaibo City Metabolic Syndrome Prevalence Study

**DOI:** 10.1155/2013/416451

**Published:** 2013-04-22

**Authors:** Valmore Bermúdez, Joselyn Rojas, Juan Salazar, Luis Bello, Roberto Áñez, Alexandra Toledo, Maricarmen Chacín, Miguel Aguirre, Marjorie Villalobos, Mervin Chávez, María Sofía Martínez, Wheeler Torres, Yaquelin Torres, José Mejías, Edgardo Mengual, Liliana Rojas, Milagro Sánchez de Rosales, Ana Quevedo, Raquel Cano, Mayela Cabrera, Rafael París, Adonías Lubo, María Montiel, Climaco Cano

**Affiliations:** ^1^Endocrine-Metabolic Research Center, “Dr. Félix Gómez,” Faculty of Medicine, University of Zulia, Maracaibo 4004, Venezuela; ^2^Institute of Clinical Immunology, University of Los Andes, Mérida, Mérida 5101, Venezuela; ^3^Endocrinology Unit, I.A.H.U.L.A, Mérida 5101, Venezuela; ^4^Institute of Biological Investigations, Faculty of Medicine, University of Zulia, Maracaibo 4004, Venezuela; ^5^Institute of Occupational Medicine, Faculty of Medicine, University of Zulia, Maracaibo 4004, Venezuela; ^6^Department of Public Health, Faculty of Medicine, University of Zulia, Maracaibo 4004, Venezuela

## Abstract

*Background*. Lipoprotein(a) [Lp(a)] is a known risk factor for cardiovascular disease, yet its influence on metabolic syndrome (MS) is still controversial. The purpose of this study was to assess the impact generated by this diagnosis in serum Lp(a) concentrations. *Materials and Methods*. A total of 1807 subjects of both genders (55.3% women and 44.7% men) belonging to the Maracaibo City Metabolic Syndrome Prevalence Study were evaluated. Results were expressed as Mean ± SD, determining differences through Student's *t*-test and One-Way ANOVA test. Multiple logistic regression models were utilized for analyzing factors associated with elevated serum Lp(a) levels and MS. Total cholesterol and LDL-C were corrected according to Lp(a)-Cholesterol when necessary. *Results*. No differences were found in Lp(a) values between genders; *P* = 0,292. The association between MS and the classification of Lp(a) was statistically significant (*χ* ^2^ = 28.33; *P* < 0,0001), with greater levels in subjects with this diagnosis. In the univariate analysis, subjects with each of the separate diagnostic criteria showed higher serum Lp(a) concentrations, except for hyperglycemia. *Conclusions*. Lp(a) values exhibit important variations regarding MS and each of its components. Impaired fasting glucose appeared as a protecting factor against elevated Lp(a) concentrations, whereas its association with LDL-C and hs-CRP suggests a potential pro-inflammatory role.

## 1. Introduction

Metabolic syndrome (MS) is a recently coined term for the designation of an aggregation of risk factors—including visceral obesity, arterial hypertension, hyperglycemia, and dyslipidemia—which in conjunction augment the probabilities of developing type 2 diabetes mellitus (DM2) and cardiovascular disease (CVD) [1]. Marquez-Sandoval et al. [2] place the prevalence of MS in Latin America at 24.9% in a previous meta-analysis. Meanwhile, in our country, the CARMELA study [3] finds the city of Barquisimeto to be parallel to Mexico City, boasting the highest prevalence of MS in Latin America in 2009. In consequence, the MS currently comprises one of the main public health issues in our territory.

Given its prominent morbidity and its importance in the ethiopathogenics of CVD, which in turn represents the main cause of mortality at a worldwide, national, and regional level [4–6], the MS has been the object of numerous investigations focused on the search of associations with new risk factors, both in general and relating to each of its specific separate components. In this sense, alterations linked to plasma lipoproteins, especially those regarding low-density lipoproteins (LDL-C) are particularly notable within the physiopathologic aspects of MS, showcasing its genetic implications [7]. Therefore, in addition to protein molecules such as high sensitivity C-reactive protein (hs-CRP), homocysteine, and fibrinogen, lipoprotein(a) [Lp(a)] represents a substantial target in the analysis of novel risk factors [8]. 

Lp(a) was initially isolated from human plasma by Berg in 1963, constituted by the association of an LDL-C particle covalently bound to a large glycoprotein, apolipoprotein(a) [Apo(a)] to apolipoprotein B by a disulfide bridge [9]. The Apo(a) chain contains five cysteine-rich domains known as “kringles”, which are coded by a gene localized in the long arm of chromosome 6 (6q26-27) and is subject to multiple polymorphisms, particularly regarding the size of kringle IV [10, 11]. In turn, this feature characterizes the different isoforms of Lp(a) and is inversely associated with plasma Lp(a) levels. These variations are outstandingly marked among races, as illustrated by the remarkably higher plasma Lp(a) concentrations in Afrodescendants [12].

Clinical interest in Lp(a) has grown exponentially in recent times, as an assortment of epidemiological studies has pinpointed the link between plasma Lp(a) concentrations (reported as ≥300 mg/L or ≥30 mg/dL) and the risk of suffering coronary events, peripheral artery disease, cerebrovascular disease, and the early development of atherosclerosis in children and adolescents [13, 14]. Despite this prominence, the interpretation and application of Lp(a) levels in clinical scenarios remain a controversial issue, since no guidelines have been suggested outlining the profiles of patients whose Lp(a) concentration should be quantified. As a result, experimental studies are required for the clarification of its role as a CVD risk factor, as well as epidemiological studies evaluating the behavior of its plasma levels regarding other CVD risk factors across different latitudes in order to effectively direct genetic studies focused on highlighting the true role of the genetic intricacies underlying the greater variations reported among demographics [15].

Stemming from this, along with the scarcity of great-scale studies detailing the epidemiological behavior of Lp(a) in Latin America, the main objective of this research was to assess the influence of its plasma levels in the MS and its individual components in adult individuals in the city of Maracaibo, Venezuela.

## 2. Materials and Methods

### 2.1. Ethical Considerations

All participants signed a written consent before being interrogated and physically examined. The study was approved by the Ethics Committee of the Endocrine and Metabolic Diseases Research Center.

### 2.2. Subjects Selection

The sample method has been already published in the Maracaibo City Metabolic Syndrome Prevalence Study cross-sectional proposal [16], yet the main aspects will be mentioned. It was a cross-sectional, descriptive, randomized, multistaged study which enrolled a total of 2,230 subjects. For this research, 1,807 subjects were studied, representing the randomly selected subsample which had their serum Lp(a) concentrations quantified.

### 2.3. Clinical Definitions

A full medical history was obtained using the Venezuelan Popular Powers Health Ministry approved medical chart filled out by trained personnel. For the measurement of blood pressure (BP), the auscultatory method was used, employing a calibrated and adequately validated sphygmomanometer. Patients were sitting and at rest for a minimum of 15 minutes, with their feet on the ground and the arm used for the measurement at the level of the heart. The procedure was performed 3 times, with 15-minute intervals. Regarding anthropometric evaluation, waist circumference values were determined employing a tape measure graduated in centimeters and millimeters (cm, mm), placing it at a point equidistant to the costal margin and the anterior superior iliac spine. For the diagnosis of MS, the criteria from the IDF/AHA/NHLBI/WHF/IASO-2009 consensus were applied [17], and American Diabetes Association criteria were used for the definition of metabolic alterations concerning glycemic status [18].

### 2.4. Laboratory Methods

Serum levels of glucose, total cholesterol, TAG, and HDL-C were determined employing commercial enzymatic-colorimetric kits (Human Gesellschaft für Biochemica and Diagnostica MBH) and specialized computerized equipment. LDL-C levels were calculated through Friedewald's formula [19], and its adjustment based on Lp(a)-bound cholesterol [Lp(a)-C] applying Dahlen's formula [LDL-C = TC − HDL-C − VLDL-C − Lp(a)-C] [20, 21]. Lp(a) was estimated through the latex turbidimetric method, Human Gesellschaft für Biochemica and Diagnostica, Germany. In this method, the presence of Lp(a) in the sample causes agglutination of latex particles coated with antibodies against Lp(a), the agglutination is proportional to the Lp(a) concentration in the sample and can be measured by turbidimetry. The cut-off value for the consideration as elevated Lp(a) levels was ≥30 mg/dL [22]. Likewise, serum hs-CRP levels were quantified employing immunoturbidimetric essays (Human Gesellschaft für Biochemica and Diagnostica MBH), and basal insulin levels were determined after 8 to 12 hours of fasting using DRG International Inc. insulin kits. For the evaluation of insulin resistance (IR), the HOMA2-IR model proposed by Levy et al. was utilized [23], determined through the HOMA-Calculator v2.2.2 program.

### 2.5. Statistical Analysis

Normal distribution of continuous variables (or lack thereof) was evaluated by using Kolmogorov-Smirnov (when *n* < 500) or Geary's (when *n* ≥ 500) test, accordingly. For normally distributed variables, the results were expressed as arithmetic mean ± SD (standard deviation). Variables without a normal distribution were logarithmically transformed, and normal distribution later corroborated. Differences between arithmetic means were assessed using Student's *t*-test (when two groups were compared) or ANOVA (when three or more groups were compared). Qualitative variables were expressed as absolute and relative frequencies, considering the results statistically significant when *P* < 0.05 in the *Z* test for proportions or *χ* ^2^ test when applied. Likewise, logistic regression models were designed, estimating odds ratios (IC 95%). The first model estimated odds ratios (ORs) for elevated Lp(a) adjusted by gender, ethnic groups, age groups, and diagnostic criteria for metabolic syndrome and hs-CRP tertiles (Tertile 1: <0.25, Tertile 2: 0.25–0.61, Tertile 3: ≥0.62 mg/L). In the second model, the same covariates were employed, with the addition of the glycemic status and LDL-C tertiles (Tertile 1: <100.67, Tertile 2: 100.67–131.99, Tertile 3: ≥132.0 mg/dL) of subjects. A third model was constructed using corrected LDL-C tertiles (Tertile 1: <93.2, Tertile 2: 93.2–123.61, Tertile 3: ≥123.62 mg/dL). Lastly, a fourth model includes risk factors for metabolic syndrome and is adjusted for gender, ethnic groups, age groups, hs-CRP tertiles, LDL-C tertiles, and Lp(a) classification by reference intervals previously reported for our population [24] and a tertile model for corrected LDL-C. The database analyses were performed using the statistical package for the social science (SPSS) v. 19 for Windows (IBM Inc. Chicago, IL, USA), considering significant results as values *P* < 0.05.

## 3. Results

### 3.1. General Characteristics of the Population

General characteristics of the studied population are presented in Table 1, while anthropometric and laboratory variables are observed in Table 2. A total of 1,807 subjects were studied, of which 55.3% (*n* = 999) belonged to the female gender and 44.7% (*n* = 808) to the male gender. The mean age was 39.2 ± 15.4 years. The mean values and percentile distribution of serum Lp(a) concentration in the general population and by gender are presented in Table 3. No differences were found when comparing males and females, resembling the behavior of HOMA2-IR, insulin, and hs-CRP concentration.

### 3.2. Lp(a) Levels and the Metabolic Syndrome

**Figure 1:**
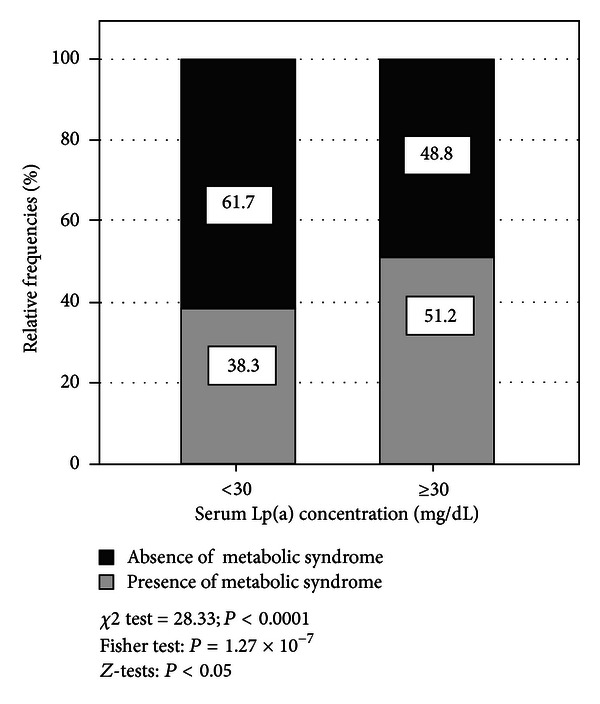
Distribution of subjects by Lp(a) categories and diagnosis of metabolic syndrome. The Maracaibo City Metabolic Syndrome Prevalence Study, 2013.

**Figure 2:**
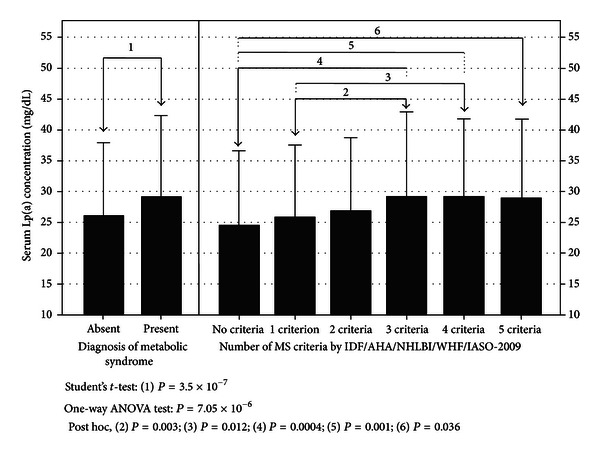
Serum Lp(a) concentration by number of criteria for the metabolic syndrome. The Maracaibo City Metabolic Syndrome Prevalence Study, 2013.

Regarding distribution of subjects with elevated Lp(a) levels, 51.2% (*n* = 339) presented a diagnosis of MS, in contrast to the proportion of individuals with normal Lp(a) levels: 38.3% (*n* = 439); *P* < 0.05. The association between the presence of MS and this lipid alteration was found to be significant (*χ* ^2^ = 28.33; *P* < 0,0001) (Figure 1). When analyzing the behavior of the serum Lp(a) concentration according to presence of MS, individuals with the diagnosis appeared to have higher levels than those without the diagnosis (with MS: 29.16 ± 13.19 versus without MS: 26.09 ± 11.84 mg/dL; *P* = 1.19 × 10^−6^). Moreover, in Figure 2 a progressive increase in Lp(a) levels was observed as the number of criteria for MS rose, with values 24.54 ± 12.07 mg/dL in subjects without any criteria, ascending to 28.95 ± 12.78 mg/dL in subjects with all criteria.

### 3.3. Lp(a) Levels and the Components of the Metabolic Syndrome

In the specific analysis of the components of MS, a similar behavior was observed for all criteria except elevated glycemia: Lp(a) concentrations were greater in subjects with each component when comparing individuals with and without each of the criteria (Table 4). Furthermore, subjects with hypertriacylglyceridemia displayed the most elevated Lp(a) levels (29.57 ± 13.02 mg/dL), and the greatest mean difference was found when comparing subjects with and without a high waist circumference. Lp(a) levels in the general population and for each gender according to the different specific diagnostic combinations for the MS are shown in Table 5. The greatest values were exhibited by subjects with the high basal glucose-low HDL-C-hypertriacylglyceridemia combination (36.96 ± 29.85 mg/dL). When comparing the means between genders, the sole statistically significant difference was found in subjects with the high waist circumference-high blood pressure-hypertriacylglyceridemia-low HDL-C combination, displaying higher serum Lp(a) concentrations in women (34.42 ± 11.69 versus 26.92 ± 11.52 mg/dL; *P* = 0.004).

### 3.4. Risk Factors for Elevated Serum Lp(a) Levels in Maracaibo

The main risk factors for presenting elevated Lp(a) concentrations were initially determined in the multivariate analysis (Table 6). In model 1, age, hypertriacylglyceridemia, hs-CRP, and elevated basal glycemia were the variables with statistical significance, where subjects aged 60 years or older presented the highest risk estimation (OR: 3.91; IC 95%: 1.97–7.76; *P* < 0.01), while elevated basal glycemia behaved as a protecting factor (OR: 0.73; IC 95%: 0.54–0.98; *P* = 0.04). Stemming from this, in model 2 the adjustment included LDL-C tertiles and the specific glycemic status of subjects, amongst which individuals with impaired fasting glucose (IFG) had the lowest risk of presenting elevated Lp(a) levels (OR: 0.69; IC 95%: 0.48–0.98; *P* = 0.04); this pattern is still observed after the adjustment of LDL-C to Lp(a)-C in the resultant tertiles. Furthermore, the main metabolic risk factors for MS are analyzed in Table 7, unveiling subjects classified in the highest LDL-C or hs-CRP tertiles to be the most associated with the diagnosis of MS, while individuals categorized in the normal interval for Lp(a) in our population displayed the lowest risk of presenting MS (OR: 0.65; IC 95%: 0.45–0.94; *P* = 0.03); after the LDL-C adjustment, the risk remains in a similar manner.

## 4. Discussion

The proportion of individuals affected by the MS worldwide shows the current pandemic magnitude of this endocrine-metabolic disorder [25], reaching prevalence figures as high as 40% in our city as contemplated by our research group (unpublished data), similar to the values obtained in this report (47%). Due to this, it has become a necessity to identify new risk factors involved in the physiopathology of MS, which may serve as predictors of its onset and as new therapeutic targets which may in turn be linked to the development of cardiovascular events [26].

As a component of MS, dyslipidemia represents one of the fundamental pillars in its ethiopathogenics, being directly related to the degree of IR and representing a series of molecular disturbances comprising the increase of the serum concentrations of apolipoprotein B, LDL-C, and VLDL-C, as well as an augmented flux of free fatty acids [27]. In the clinical setting, these disorders translate into the widely known criteria for elevated TAG and low HDL-C [28]. Furthermore, these lipid alterations are intimately associated with a chronic inflammatory state, which represents the essential mechanism from which atherosclerosis and CVD stem [29, 30].

Based on these premises, dyslipidemia, and inflammation, Lp(a) plays an important role at the molecular level both for CVD and MS when its plasmatic concentration is elevated, being able to generate both of the aforementioned basic disturbances [31–33]. However, research assessing its epidemiological behavior remains scarce. A great deal of these studies have been executed in European and Asian populations, showing proportions of individuals with MS and high Lp(a) similar to ours, with prevalence figures as elevated as 51.4% in a small Turkish study [34].

It is important to highlight the lack of differences of Lp(a) levels between genders in this report, as has been outlined in previous investigations [31, 35]; therefore, most comparisons were done utilizing the general population. Exhibiting a qualitative association with Lp(a), subjects with MS also showed higher levels than healthy subjects, similar to the results of Bozbaş et al. [34], in 355 Turkish individuals. Nevertheless, this behavior differs from that described for older Japanese adults, whose plasmatic concentrations were not statistically different [36]. Notably, notwithstanding the escalating tendency of Lp(a) levels as the number of criteria increased, it is not the amount of criteria expressed but the actual diagnosis of MS that appears relevant regarding the presence of elevated Lp(a) concentrations.

With reference to the analysis by individual diagnostic criteria, previous studies evaluating the relationship between Lp(a) and the isolated components MS are not abundant, and very few include all criteria in their analyses [37–40]. In our univariate estimations, subjects displaying each of the components appeared to have higher serum Lp(a) concentrations in contrast to those without these conditions, except those with elevated glycemia, where differences were not statistically significant. These results differ from those depicted by Cândido et al. [41] in 400 Brazilian individuals, who did not find such association with these criteria in an analysis akin to ours. It is important to acknowledge that the variables demonstrating the greater differences in Lp(a) levels (waist circumference and elevated TAG) are the most associated with systemic inflammatory state characteristic of MS [42, 43]. These findings may underline the role of Lp(a) in this process, whether as an active molecule or as a potential proinflammatory “companion” of these risk factors [44, 45]. 

Likewise, when assessing its plasmatic concentration according to the possible specific diagnostic combinations for MS criteria, a large heterogeneity was found concerning these levels and the amount of criteria; yet, the greatest values were found in subjects with more than 3 alterations. Notoriously, the high basal glucose/low HDL-C/hypertriacylglyceridemia combination displayed the highest Lp(a) values, and females only showed larger figures only within the subset of subjects with high waist circumference/high blood pressure/hypertriacylglyceridemia/low HDL-C combination; in addition, these women also had higher LDL-C levels. These phenomena turn both of these groups of patients into potential candidates for the application of therapeutic measures aimed to the decrease of Lp(a) values, particularly with an increment in the degree of physical activity performed, since it has been associated with normal levels of this lipoprotein in our demography [46]. These patients are also ideal candidates for the investigation of genetic disorders which may be responsible for this dyslipidemia [47].

Indeed, the decisive role played by genetic factors regarding Lp(a) is broadly known [31, 48]; nonetheless, several conditions, alterations, and molecules can influence and generate important variations in its plasmatic concentration [49]. In our population, age appears to be one of the main risk factors for presenting elevated Lp(a), resembling previous reports on the Taiwanese population [50] and on Swedish subjects from the MONICA study [51]. Moreover, despite the cardiovascular consequences generated by high levels of this molecule, when it coexists with specific Apo(a) isoforms, it has been associated with longevity [52]. 

On the other hand, in the multivariate analysis of all diagnostic criteria for MS, only patients with hypertriacylglyceridemia exhibited a greater risk of presenting elevated Lp(a). However, after adjusting the model for LDL-C categories, not only is it apparent that this lipoprotein boasts the closest association with high levels of Lp(a), but the effects of TAG seem to disappear; it is important to highlight that this tendency was only observed with LDL-C adjusted for Lp(a)-C, not priorly. This pattern deviates from those portrayed by rainwater in healthy subjects [53] and Hernández et al. in diabetic patients [54], who both found a positive (Lp(a) − LDL-C) relationship and an inverse (Lp(a) − TAG) relationship. Therefore, future studies should focus on the evaluation of the behavior of Lp(a) with respect to the various types of dyslipidemia, the understanding of molecular mechanisms explaining the proportionality of LDL-C/Lp(a) concentrations, and the therapeutic considerations that may be established for these patients [55].

Another relevant finding was the “protective” property displayed by elevated glycemia, a complex MS diagnostic criterion which required further more detailed categorization due to its overwhelming heterogeneity (Table 6, Model 3). Subjects with IFG yielded a lower risk (29%) of presenting elevated Lp(a) values in comparison to normoglycemic individuals. This behavior is intimately linked to the impact of insulin in the metabolism of Lp(a), where it has been attributed an inhibiting effect in the synthesis of Apo(a) in animal models [56], supported by inverse relationships observed between both molecules in population studies [57, 58]. Of all glycemic status subgroups, subjects with IFG presented the most augmented values of insulinemia, statistically different to those of the normoglycemics (19.23 ± 12.84 versus 14.17 ± 8.45 mg/dL; *P* < 0.05). Despite the fact that the group of diabetics showed high levels of insulin (18.93 ± 12.58), its effect may have been attenuated due to their inferior beta cell functionality and higher levels of IR when compared to subjects who only presented IFG. Although few studies have shown an inverse relationship between Lp(a) concentration and the presence of DM [59, 60], such an association has not been reported in the context of a premorbid state.

Another interesting finding from this study is that the subjects with the highest hs-PCR and Lp(a) levels were the ones obtaining the highest cardiovascular risk, which could be attributed to the inflammatory properties that both molecules have [31, 44]. Even though the particular characteristics of hs-PCR have been previously characterized in our population [61], other investigations should be undertaken to properly evaluate the interaction between these two.

Finally, when exploring the factors that exhibited the greatest association with the diagnosis of MS, subjects with high LDL-C and hs-CRP displayed the most substantial risk of presenting it. Concerning the dyslipidemia, up to 1.8 times more risk was ascertained in individuals with values higher than 132 mg/dL, confirming the position performed by these molecules in the physiopathology of MS; even after the adjustment of LDL-C, the risk of presenting MS is similar (OR: 1.7). In this light, it becomes relevant to determine the proportion of LDL-C that is already oxidized, as it may unveil the link between MS and CVD, since they are considered powerful inflammatory products [62]. At any rate, regardless of the lipid phenotype, pharmacological management remains fundamental in these patients [63]. With respect to elevated hs-CRP values, findings were similar, albeit exhibiting a greater risk: 2.4 times more probability of developing MS, showcasing the elementary inflammatory component underlying MS and the independent effect of this protein in relation to other risk factors [64, 65]. Notably, despite Lp(a) not being related to higher risk of MS as its concentration increased, individuals classified in the normal interval of  Lp(a) by reference values specific to our population [24] depicted a lower risk of developing MS when adjusted by other inflammatory factors. This reinforces the importance of each of these metabolic disturbances in the integral management of subjects in risk and patients with MS. However, this is a cross-sectional study, which makes it difficult to make decisions concerning causality.

This analysis demonstrates that MS is yet another disease to consider among disorders involving high Lp(a) levels; future studies are required for discerning whether this relationship represents a state previous to the widely recognized cardiovascular consequences of this molecule, or if they each stand as independent outcomes. Likewise, the presence of MS influences the plasmatic concentration of Lp(a), but this effect is irrespective of the amount of diagnostic criteria collected once the individual is ill. Although these criteria seem to modify levels when they are present, when assessed in conjunction, their effects appear to be attenuated. The only component to show an association despite several statistical adjustments is impaired fasting glucose, which, by virtue of being related to a hyperinsulinemic state, appears to diminish the probability of presenting elevated Lp(a), an association that had previously only been suggested for DM2.

## Figures and Tables

**Table 1:** General characteristics of the population evaluated by gender. The Maracaibo City Metabolic Syndrome Prevalence Study, 2013.

	Females		Males		Total	
	*n*	%	*n*	%	*n*	%
Age group (%)						
18-19	86	8.6	71	8.8	157	8.7
20–29	221	22.1	246	30.4	467	25.8
30–39	175	17.5	138	17.1	313	17.3
40–49	233	23.3	147	18.2	380	21.0
50–59	169	16.9	125	15.5	294	16.3
≥60	79	7.9	55	6.8	134	7.4
Ethnic group (%)						
Mixed race	728	72.9	594	73.5	1322	73.2
Hispanic Whites	173	17.3	141	17.5	314	17.4
Afro-Venezuelans	29	2.9	33	4.1	62	3.4
American-Indians	58	5.8	39	4.8	97	5.4
Others	11	1.1	1	0.1	12	.7
Metabolic syndrome (%)^¶^						
Absent	601	60.2	428	53.0	1029	56.9
Present	398	39.8	380	47.0	778	43.1
High waist circumference (%)^¶^						
Absent	220	22.0	232	28.7	452	25.0
Present	779	78.0	576	71.3	1355	75.0
High blood pressure (%)^¶^						
Absent	658	65.9	439	54.3	1097	60.7
Present	341	34.1	369	45.7	710	39.3
High basal glycemia (%)^¶^						
Absent	733	73.4	526	65.1	1259	69.7
Present	266	26.6	282	34.9	548	30.3
Low HDL-C (%)^¶^						
Absent	354	35.4	405	50.1	759	42.0
Present	645	64.6	403	49.9	1048	58.0
High triacylglycerides (%)^¶^						
Absent	782	78.3	545	67.5	1327	73.4
Present	217	21.7	263	32.5	480	26.6
Lp(a) classification (%)						
<30 mg/dL	631	63.2	514	63.6	1145	63.4
≥30 mg/dL	368	36.8	294	36.4	662	36.6

Total (%)	999	55.3	808	44.7	1807	100.0

^¶^IDF/AHA/NHLBI/WHF/IASO-2009.

**Table 2:** Clinical and biochemical parameters evaluated by gender. The Maracaibo City Metabolic Syndrome Prevalence Study, 2013.

	Females (*n* = 999)	Males (*n* = 808)	P*
	Mean ± SD	Mean ± SD	
Age (years)	40.1 ± 15.3	38.1 ± 15.5	0.003
Waist circumference (cm)	91.0 ± 14.1	98.8 ± 15.9	1.73 × 10^−27^
Basal glycemia (mg/dL)	98.5 ± 31.2	101.4 ± 33.8	0.018
Insulin (UI/mL)	15.1 ± 9.7	15.6 ± 10.0	0.729
HOMA2-IR	2.3 ± 1.4	2.4 ± 1.5	0.540
TAG (mg/dL)	115.1 ± 85.4	142.9 ± 116.5	3.83 × 10^−11^
Total cholesterol (mg/dL)	192.0 ± 43.5	185.9 ± 47.8	0.001
Corrected total cholesterol (mg/dL)	183.7 ± 43.0	177.8 ± 47.6	0.001
HDL-C (mg/dL)	46.9 ± 11.8	41.2 ± 11.8	8.77 × 10^−28^
LDL-C (mg/dL)	122.1 ± 37.0	117.1 ± 39.1	0.001
Corrected LDL-C (mg/dL)	113.8 ± 36.5	109.2 ± 38.8	0.004
SBP (mmHg)	117.2 ± 17.0	122.9 ± 15.9	1.18 × 10^−14^
DBP (mmHg)	75.4 ± 10.8	79.3 ± 11.5	1.32 × 10^−13^
hs-CRP (mg/L)^‡^	0.40 (0.16–0.84)	0.43 (0.20–0.74)	0.387

*Student's *t*-test (after logarithmic transformation).

^‡^Values expressed as median (interquartile range). Comparison: Mann-Whitney's *U* test.

SD: standard deviation; TAGs: triacylglycerides; HDL-C: high-density lipoprotein; LDL-C: low-density lipoprotein; SBP: systolic blood pressure; DBP: diastolic blood pressure; hs-CRP: high sensitivity C-reactive protein.

**Table 3:** Mean values and percentile distribution of serum Lp(a) concentrations in the general population and by gender. The Maracaibo City Metabolic Syndrome Prevalence Study, 2013.

	Serum Lp(a) concentration (mg/dL)						
	Mean*	SD	p05th	p25th	p50th	p75th	p95th
Females	27.69	12.13	8.90	19.70	26.60	35.10	50.00
Males	27.07	13.00	7.00	18.45	25.50	35.30	51.60

Total	27.41	12.53	8.00	19.20	26.20	35.20	50.80

*Student's *t*-test: *P* = 0.292.

**Table 4:** Serum Lp(a) concentration assessed by criteria for the metabolic syndrome. The Maracaibo City Metabolic Syndrome Prevalence Study, 2013.

MS criteria^‡^	Lp(a) (mg/dL)	
	Mean	SD
High waist circumference		
Absent	24.99	12.19
Present	28.22	12.54
P*	1.93 × 10^−6^	
High blood pressure		
Absent	26.29	12.20
Present	29.15	12.83
P*	2.5 × 10^−6^	
High basal glycemia		
Absent	27.61	12.03
Present	26.95	13.62
P*	0.328	
Low HDL-C		
Absent	26.51	12.81
Present	28.07	12.29
P*	0.009	
High TAG		
Absent	26.63	12.26
Present	29.57	13.02
P*	1.83 × 10^−5^	

MS: metabolic syndrome; SD: standard deviation; TAGs: triacylglycerides.

^‡^Defined by IDF/AHA/NHLBI/WHF/IASO-2009.

*Student's *t*-test.

**Table 5:** Serum Lp(a) concentration in the general population and by gender according to specific diagnostic combinations for the metabolic syndrome. The Maracaibo City Metabolic Syndrome Prevalence Study, 2013.

Metabolic syndrome	Number of criteria for MS			Lp(a) (mg/dL)						
			*n*	Females		Males		Total		P*
				Mean	SD	Mean	SD	Mean	SD	
Without MS	No criteria	Healthy	**185**	**24.79**	**11.63**	**24.31**	**12.50**	**24.54**	**12.07**	**0.790**
	1 criterion	W	189	26.90	12.25	26.47	11.20	26.72	11.80	0.806
		B	20	22.55	8.27	26.74	10.32	26.32	10.03	0.589
		G	27	25.99	11.31	18.23	13.19	20.53	12.95	0.159
		H	122	25.03	11.15	26.33	10.76	25.46	10.99	0.538
		T	9	30.23	14.37	27.52	15.76	28.42	14.45	0.810
	2 criteria	WB	103	26.23	11.74	29.47	12.47	27.80	12.15	0.177
		WG	52	24.41	12.03	26.25	15.08	25.12	13.17	0.628
		WH	233	27.45	10.37	25.94	13.08	27.14	10.97	0.397
		WT	16	22.12	12.65	31.74	11.83	28.73	12.54	0.162
		BG	14	34.45	8.67	18.82	14.32	23.29	14.59	0.067
		BH	19	26.50	8.32	21.54	11.59	23.37	10.54	0.337
		BT	0	—	—	—	—	—	—	—
		GH	20	26.75	16.49	23.34	16.71	25.56	16.21	0.666
		GT	4	—	—	20.42	11.78	20.42	11.78	—
		HT	16	30.21	8.66	32.28	10.83	31.24	9.53	0.680
With MS	3 criteria	WBG	46	33.49	13.47	26.21	14.56	28.90	14.46	0.099
		WBH	133	30.61	12.73	32.26	13.27	31.17	12.89	0.487
		WBT	38	34.78	13.26	32.11	14.32	32.88	13.90	0.598
		WGH	65	23.82	13.99	21.53	12.79	22.90	13.47	0.506
		WGT	17	25.49	17.56	27.33	16.08	26.57	16.18	0.826
		WHT	92	28.67	11.12	31.55	13.12	29.99	12.09	0.257
		BGH	2	—	—	25.89	2.52	25.89	2.52	—
		BGT	5	33.75	6.43	23.83	15.82	27.80	12.85	0.478
		GHT	4	28.81	30.64	61.40	—	36.96	29.85	—
		BHT	2	—	—	16.85	20.58	16.85	20.58	—
	4 criteria	WBGH	97	28.52	11.83	28.66	12.45	28.58	12.06	0.957
		WBGT	34	32.16	17.14	27.70	15.35	29.01	15.77	0.461
		WBHT	82	34.42	11.69	26.92	11.52	30.58	12.14	0.004
		BGHT	3	29.60	—	24.30	9.05	26.07	7.09	—
		WGHT	46	29.23	10.48	27.36	14.23	28.34	12.31	0.612
	5 criteria	All	**112**	**29.20**	**13.62**	**28.73**	**12.09**	**28.95**	**12.78**	**0.846**

W: high waist circumference; B: high blood pressure; G: high basal glucose; H: low HDL-C; T: high TAG.

*Student's *t*-test.

**Table 6:** Logistic regression models of risk factors for high serum Lp(a) concentration. The Maracaibo City Metabolic Syndrome Prevalence Study, 2013.

	Model 1*				Model 2**		Model 3***	
	Crude odds ratio (CI 95%^a^)	*P* ^b^	Adjusted odds ratio (CI 95%^a^)	*P* ^b^	Adjusted odds ratio (CI 95%^a^)	*P* ^b^	Adjusted odds ratio (CI 95%^a^)	*P* ^b^
Age group (years)								
<20	1.00	—	1.00	—	1.00	—	1.00	—
20–29	1.18 (0.77–1.80)	0.46	1.28 (0.72–2.27)	0.40	1.21 (0.68–2.15)	0.53	1.27 (0.71–2.25)	0.42
30–39	1.72 (1.11–2.67)	0.02	1.86 (1.01–3.45)	0.05	1.58 (0.85–2.95)	0.15	1.78 (0.96–3.32)	0.07
40–49	2.78 (1.82–4.24)	<0.01	2.97 (1.63–5.43)	<0.01	2.51 (1.36–4.64)	<0.01	2.83 (1.53–5.21)	<0.01
50–59	2.42 (1.56–3.75)	<0.01	2.78 (1.48–5.22)	<0.01	2.14 (1.12–4.08)	0.02	2.54 (1.34–4.82)	<0.01
≥60	3.80 (2.38–6.06)	<0.01	3.91 (1.97–7.76)	<0.01	3.40 (1.67–6.91)	<0.01	3.93 (1.94–7.96)	<0.01
High waist circumference (%)^¶^								
Absent	1.00	—	1.00	—	1.00	—	1.00	—
Present	1.77 (1.40–2.23)	<0.01	0.89 (0.62–1.27)	0.52	0.82 (0.56–1.18)	0.28	0.89 (0.62–1.28)	0.53
High blood pressure (%)^¶^								
Absent	1.00	—	1.00	—	1.00	—	1.00	—
Present	1.64 (1.35–1.99)	<0.01	1.24 (0.92–1.65)	0.15	1.23 (0.91–1.65)	0.18	1.24 (0.92–1.67)	0.16
Low HDL-C (%)^¶^								
Absent	1.00	—	1.00	—	1.00	—	1.00	—
Present	1.22 (1.00–1.48)	0.05	1.07 (0.82–1.41)	0.61	1.12 (0.85–1.48)	0.43	1.09 (0.82–1.43)	0.56
High TAG (%)^¶^								
Absent	1.00	—	1.00	—	1.00	—	1.00	—
Present	1.54 (1.25–1.91)	<0.01	1.37 (1.01–1.86)	0.05	1.30 (0.95–1.79)	0.10	1.38 (1.00–1.88)	0.05
hs-CRP (mg/L)								
<0.25	1.00	—	1.00	—	1.00	—	1.00	—
0.25–0.61	1.10 (0.81–1.50)	0.53	1.03 (0.75–1.41)	0.87	1.01 (0.73–1.40)	0.96	1.02 (0.74–1.40)	0.92
≥0.62	1.75 (1.30–2.36)	<0.01	1.48 (1.08–2.04)	0.02	1.55 (1.12–2.14)	<0.01	1.52 (1.10–2.09)	0.01
High basal glycemia^¶^								
Absent	1.00	—	1.00	—	—	—	—	—
Present	1.06 (0.86–1.31)	0.58	0.73 (0.54–0.98)	0.04	—	—	—	—
Glycemic status								
Normoglycemia	1.00	—	—	—	1.00	—	1.00	—
Impaired fasting glucose	0.96 (0.76–1.21)	0.71	—	—	0.69 (0.48–0.98)	0.04	0.71 (0.50–0.99)	0.05
Type 2 diabetes mellitus	1.37 (0.98–1.93)	0.07	—	—	0.67 (0.41–1.10)	0.11	0.66 (0.41–1.07)	0.09
LDL-C (mg/dL)								
<100.67	1.00	—	—	—	1.00	—	—	—
100.67–131.99	1.43 (1.11–1.83)	<0.01	—	—	1.31 (0.93–1.84)	0.12	—	—
≥132.0	2.21 (1.74–2.82)	<0.01	—	—	2.09 (1.49–2.93)	<0.01	—	—
Corrected LDL-C (mg/dL)								
<93.2	1.00	—	—	—	—	—	1.00	—
93.2–123.61	1.11 (0.87–1.42)	0.39	—	—	—	—	0.94 (0.67–1.31)	0.70
≥123.62	1.42 (1.12–1.80)	<0.01	—	—	—	—	1.20 (0.86–1.67)	0.28

^¶^IDF/AHA/NHLBI/WHF/IASO-2009.

^a^Confidence interval (95%). ^b^Level of significance.

*Model 1: Adjusted by gender, ethnic group, and age group. Diagnostic criteria for metabolic syndrome and hs-CRP tertiles.

**Model 2: similar adjustment, with the addition of specific glycemic status and LDL-C tertiles.

***Model 3: similar adjustment, with corrected LDL-C tertiles.

**Table 7:** Logistic regression model of risk factors for the metabolic syndrome. The Maracaibo City Metabolic Syndrome Prevalence Study, 2013.

	Model 1*				Model 2**	
	Crude odds ratio (CI 95%^a^)	*P* ^b^	Adjusted odds ratio (CI 95%)	*P*	Adjusted odds ratio^c^ (CI 95%)	*P*
Lp(a) (mg/dL)						
<18.40	1.00	—	1.00	—	1.00	—
18.40–33.84	0.97 (0.76–1.24)	0.82	0.65 (0.45–0.94)	0.03	0.67 (0.46–0.97)	0.03
≥33.85	1.73 (1.33–2.26)	<0.01	0.76 (0.50–1.14)	0.19	0.82 (0.54–1.23)	0.33
hs-CRP (mg/L)						
<0.25	1.00	—	1.00	—	1.00	—
0.25–0.61	1.18 (0.87–1.60)	0.28	1.02 (0.72–1.44)	0.93	1.02 (0.72–1.44)	0.92
≥0.62	2.62 (1.95–3.53)	<0.01	2.47 (1.75–3.49)	<0.01	2.47 (1.75–3.48)	<0.01
LDL-C (mg/dL)						
<100.67	1.00	—	1.00	—	—	—
100.67–131.99	1.92 (1.51–2.46)	<0.01	1.59 (1.11–2.28)	0.01	—	—
≥132.0	3.24 (2.54–4.13)	<0.01	1.81 (1.26–2.59)	<0.01	—	—
Corrected LDL-C (mg/dL)						
<93.2	1.00	—	—	—	1.00	—
93.2–123.61	1.75 (1.37–2.23)	<0.01	—	—	1.51 (1.06–2.16)	0.02
≥123.62	3.00 (2.36–3.81)	<0.01	—	—	1.71 (1.20–2.43)	<0.01

^a^Confidence interval (95%). ^b^Level of significance.

*Adjusted by gender, ethnic group, age group, Lp(a) classification, hs-CRP tertiles, and LDL-C tertiles.

**Adjusted by gender, ethnic group, age group, Lp(a) classification, hs-CRP tertiles, and corrected LDL-C tertiles.
